# Clinical Notes upon the Use of the Galvano-Cautery

**Published:** 1880-01

**Authors:** William A. Byrd

**Affiliations:** No. 407 Jersey Street; Quincy, Illinois


ARTICLE IV.

### CLINICAL NOTES UPON THE USE OF THE GALVANO-CAUTERY.

BY WILLIAM A. BYRD, M. D., ETC., QUINCY, ILLINOIS.

The galvano-cautery, as a safe and reliable means of performing many operations, has not been appreciated by the profession generally to the extent that its merits demand. That a more frequent use may be made of it is the hope that actuates me to record a few of the cases illustrating its use in my hands.

*Follicular Pharyngitis and Nasal Catarrh.*—Early in June, 1878, Prof. W. L. P. C——, aged 35, consulted me in regard to follicular pharyngitis and nasal catarrh that he had been troubled with for years.

He had been under the treatment of some excellent physicians, but never obtained any but temporary relief. The nasal mucous membrane was red and swollen so as to seriously interfere with breathing. The pharynx was granular, with dilated and tortuous vessels in the mucous membrane. The voice husky, and impaired further by the partial closure of the nostrils.

June 29th, 1878, with the assistance of Drs. M. Rooney, W. C. Pipino, and J. A. Wagner, with the patient under chloroform, I applied the wire of the galvano-cautery battery to the posterior pharynx, making three parallel applications an inch and a half long, an inch apart, burning just the width of the wire and barely through the mucous membrane. I also cauterized the mucous membrane over the middle turbinated bone in each nostril pretty thoroughly. A gargle of a weak solution of chlorate of potassa with carbolic acid was ordered. The nostrils to be syringed out with warm salt water as the occasion required. The next morning there was considerable swelling and pain, which rapidly subsided. In a week he was well, and has remained so.

The contraction of the cicatricial tissue deprived the mucous membrane of the nostrils of its erectile property and obliterated the enlarged varicose vessels of the pharynx.

Since then I have operated in the same manner upon a good many patients with uniformly good results in all but two, so far as I know. One of the two left the city before I could determine whether she was well or not; the other got well of the pharyngitis and catarrhal trouble in one nostril. The other nostril was obstructed by a deflection of the septum narium, that I expect to remedy by an operation suggested by Dr. A. J. Steele, of St. Louis, and published in the St. Louis *Courier of Medicine*, for May, 1879 (p. 485, *et seq.*), an operation by the performance of which I very effectually relieved Dr. Jacob A. Wagner—mentioned above—of the same trouble.

*Fistula in Ano and Hemorrhoides.*—In the treatment of these affections the galvano-cautery excels anything that I have made use of after dilatation of the rectum. For my opinions expressed at greater length upon this subject, I beg leave to refer the reader to an article of mine, entitled "Dilatation of the Rectum in the Treatment of Hemorrhoides, Fissured Anus, and as a Prophylactic Measure in Fistulæ," published in the May 24th, 1879, number of the *Hospital Gazette*, and the August 29th, 1879, number of the *Medical Press and*

*Circular.* Its superiority over the ligature is its causing less pain and the short time required to perform the operation in its entirety. In operating for fistulæ by this means there is freedom from pain and hemorrhage, and an eschar is left to hold the wound open and secure union from the bottom, obviating the liability to bridging over of tissue and the re-establishing of the trouble, as is the case if the patient is operated upon with the knife and the after-treatment entrusted to careless or incompetent hands.

George L——, a middle-aged bartender, presented himself with a fistula in ano of fifteen years' standing. With the assistance of Dr. Wm. C. Pipino, a platinum wire was passed through the fistula, and the current passed, when the heated wire was drawn slowly through the tissues, causing but little pain and no hemorrhage. No anæsthetic was used. A cherry stone, and a bit of wood nearly an inch long and half an inch wide, were found in the fistulous track. The wound was dressed with an oiled rag pressed down to the bottom of the wound, and renewed night and morning. No anodynes were required in the after-treatment. In ten days he was well.

August 1st, 1879, was called to see Miss Mary S——, aged 22, who was suffering from dysmenorrhea and hemorrhoides. The dysmenorrhea was the result of a sharp ante-flexion of the uterus. There was habitual constipation, which likely produced the piles in the first instance, which condition being established contributed to keep up the constipation. Defecation being painful caused the patient to retain the contents of the bowels as long as possible; the mass, becoming harder and larger, distended the rectum and pressed the uterus out of position, interfering with the pelvic circulation, which again deranged the nutrition of the uterus and aggravated the hemorrhoides.

I first dilated the uterus with laminaria tents, and August 6th, with the assistance of Dr. M. Rooney, the patient having been put under the influence of ether, I dilated the rectum fully and removed the hemorrhoides with Smith's clamp and the caustic wire. The pain after the operation was so slight as to require but one dose of one-fourth of a grain of morph. sulph. for its relief. In a week she was well, and has remained so.

*Epithelioma and Rodent Cancer.*—August 22d, 1878, Mrs. George C——, aged 40, called with her husband to consult me in regard to a wart-like excrescence, about the size of a walnut, that had made its appearance as a small nodule, some two years before, upon the outer side of the left breast. Without an anæsthetic, and with the assistance of her husband, I passed the wire around the tumor about half an inch external to its base, which I was enabled to do by making traction upon the outer portion of the tumor, thereby raising the base and surrounding skin up into a cone shape. Passing the current, and keeping up the traction, the outer side was cut through first, leaving the traction applied to the central attachments. By this procedure a cone-shaped excavation was left at the site of the tumor. The wound was dressed with carbolized vaseline and oakum. She stated that the pain was insignificant. Recovery was speedy and complete.

March 13th, 1879, I was called to Edina, Missouri, by Dr. Lewis F. Nelson, to see Andrew B——, aged 48, who was suffering from epithelioma of the left lower lip. At the corner of the mouth a place was eaten away as large as the end of the index finger, through which the saliva dribbled. The edges of the ulcer were creviced, red, angry looking and discharging ichor.

A platinum wire was passed through the cheek with a Darby's perineum needle, opposite the corner of the mouth, and half an inch external to the nearest discoverable diseased tissue; the current was passed and the wire was gradually drawn upon, keeping carefully half an inch within the sound tissue until it cut out a little to the right of the centre of the lower lip. The loop was then placed at the point of commencement and carried around half an inch within the sound tissue, coming out a little to the left of the middle of the upper lip. The piece taken out was about the size of my thumb. An iron wire suture was then passed, about half an inch from the edge of the opening, through the whole thickness of the lip, and a leaden shield and perforated shot clamp put upon either end so as to draw the edges of the wound together. When the eschar was thrown off by suppuration, the granulations of either side were held in contact by this stay-suture and united, leaving, as Dr. Nelson writes me, a healthy lip, with very little deformity, and capable of retaining the saliva.

September 13th, 1879, Mrs. Paul K——, aged 56, consulted me in regard to a rodent cancer of the left ala, of ten years' standing. The ulcer was about the size of a silver quarter dollar, only the outlines were irregular and the edges overhanging. Plate III., figure 2, fasciculus I., *Hutchinson's Clinical Surgery*, is an excellent representation of the condition, with the exception that Hutchinson figures the ulcer upon the right nostril. It began first as a spot of enlarged blood vessels, over which the skin thinned and gave way, permitting an ichorous discharge that would sometimes become dry enough to make gray, brittle, light-colored scab. Various caustic applications were applied by the physicians she consulted, but did no good, the sore continually but very gradually enlarging and giving considerable annoyance from the discharge and the itching, and also from the inquiries of anxious friends about her red sore nose. I applied the heated wire to the whole surface of the ulcer and for some distance around it, destroying all the tissue I thought to be diseased. The wound was dressed with carbolized vaseline. She did not complain of pain during the operation. In ten days she was well.

February 8th, 1879, I saw, with Dr. J. A. Wagner, Mrs. Julia B——, aged 60, who was suffering with epithelioma, involving the whole gum of the right upper jaw. Owing to mitral insufficiency we did not give an anæsthetic, and the patient would permit only a partial removal of the diseased tissue. The operation gave no relief. This was the only patient that I have operated on that complained of much pain from the cautery.

*Aneurism by Anastomosis.*—October 6th, 1878, Dr. P. A. Marks brought to me Prof. C——, aged 36, who was suffering with a "mother's mark" in the right hypochondriac region. In infancy it was very small; of late it had grown rapidly until it was the size of a hickory nut when seen by me. It had been irritated by the rubbing of the waistband of his trowsers, and was considerably inflamed. The platinum loop was thrown around its base, and the current passed. Very gradual traction was made upon the wire, so as to cut very slowly and cauterize well as it divided the tissues. The operation was almost painless and perfectly bloodless. Recovery was rapid.

*Tracheotomy.*—For my experience with the galvano-cautery in the performance of this operation, I beg leave to refer the reader to an article upon that subject in the November, 1879, number of the St. Louis *Clinical Record*, or will send any one a reprint of it who may desire it.

*Extirpation of the Rectum.*—Mrs. Philip B——, aged 61, was sent to me by Dr. S. W. Durant, of Adams, Illinois, to have an operation performed to give her relief from pain, loss of blood and tenesmus during defecation. I sent her to St. Mary's Hospital. An examination revealed, an inch and a half up the rectum, an indurated, nodulated mass, of half an inch or more in thickness, occupying the bowel and infiltrated in its coats for two inches. The opening through this mass would barely admit the passage, with some force, of the index finger, and was ulcerated throughout. From the ulcerated surfaces came a good deal of pus and blood when hardened feces were forced through the small opening. Dr. Durant was of the opinion that the stricture was of a malignant nature.

I determined to remove the strictured portion of the bowel with the curette and the caustic wire, through dilating the sphincters, thereby saving those muscles for future use.

February 25th, 1879, with the assistance of Drs. J. F. Durant, M. Rooney, M. Renick and J. A. Wagner, with the patient under ether, I introduced my hand, as a divulsor, into the rectum and through the stricture. The rectum was then held open with wire loop specula, while I scraped the softest portions of the growth with a Sims' sharp uterine curette. The hard nodules were lifted with forceps and held terse while the heated wire was thrown around and cut through below their bases. All points where blood issued were well cauterized. There was great hemorrhage while using the curette.

Warm injections of a solution of chlorate of potassa were ordered every four hours as an antiseptic. Anodynes were given as necessary, though she required but little, and tonics.

Large-sized rectal bougies were passed daily after the first week, to prevent the cicatrix from contracting so as to reproduce a stricture.

March 25th, there being no ulceration nor discharge of pus or blood or tenesmus, she was discharged. The cicatricial tissue that occupied the place of the tumor was not more than half an inch wide, was smooth, elastic, and had an opening through it of an inch and a half diameter.

The tissues of the constricting mass were carefully examined under the microscope, but no evidence of malignancy was discovered.

May 23d, she returned, complaining of difficulty in passing feces if hardened, but free from hemorrhage or suppuration. The cicatricial tissue had contracted, leaving the calibre of the bowel not more than three quarters of an inch in diameter. This, with the assistance of Dr. S. W. Durant, I dilated, under ether, with my hand, and ordered it to be kept open by the daily use of Wales' soft rubber dilators.

August 1st, she returned, with constipation and trouble higher up the bowel, beyond my reach. With laxatives and tonics she improved, and is doing fairly well now; yet I believe, that although we were unable to determine carcinoma, that disease is the trouble, and that she will yet die of it.

*Oozing Tumor of the Labia.*—Mary E. C——, aged 30, consulted me in regard to lobulated, raspberry-like excrescences upon both labia, extending down nearly to the anus. These growths rose above the surrounding skin, from one-half to three-quarters of an inch, and constantly exuded a stinking watery ichor that almost drove the woman wild with pain. I prescribed washes of solutions of carbolic acid of different strength, carbolic acid and glycerine, solutions of chloral, dry oxide of zinc, subnitrate of bismuth, and everything else that I thought would possibly give her relief. Nothing did any good.

September 28th, 1878, with the assistance of Dr. William Zimmerman, I removed the growths with the galvano-caustic loop, and cauterized the parts from whence they were removed deeply. The wound was dressed with carbolized vaseline. Recovery was rapid and complete.

*Amputation of the Cervix Uteri.*—Mrs. Jerome M——, aged 35, came to me for relief from dysmenorrhea and dyspareunia. In making an examination, the upper portion of the vagina was found occupied by a tumor smooth to the touch, somewhat larger than a hen's egg, and reaching to within an inch of the vulva. This could hardly be a polypus, as there had been no metrorrhagia, neither could it be an inverted uterus, as the lady was nulliparous, and besides I could detect the body of the uterus in its proper place by conjoined palpation. Introducing a speculum I was still more at a loss, for I discovered a smooth, light pink, oval mass of about the diameter of the bottom of a quinine bottle between the blades of the speculum, but no where could I see an os. It being a gloomy day and late in the afternoon, I told her I had not fully decided what was the matter, but to call again earlier in the day.

At her next visit I detected a spot of mucus upon the centre of the tumor, and wiping it away discovered an opening not larger than the end of a knitting needle. Exploring this with a small probe I found it to be the os. The uterus was in its normal position but was four inches and a half deep. Here, then, was a case of hypertrophy of the cervix causing mechanical dysmenorrhea by encroaching upon the cervix, and dyspareunia by encroaching upon the vaginal space.

Amputation of the cervix offered the only hope to her for recovery, and Sept. 30th, 1878, assisted by Drs. M. F. Bassett, M. Rooney and J. A. Wagner, the patient under ether and in Sims' position, the vagina held open with a Sims speculum and the cervix steadied with a vulsellum, I threw the wire around the cervix one inch from its extremity. One end of the wire was screwed tightly in the conducting rod on one side of the handle and passed through a hole I had drilled in the conductor on the other side so as to allow it to draw through easily, at the same time not permit the circuit to be broken Then, by passing the current and drawing upon the loose end of the wire with a pair of small pliers, so as to have it cut slowly through the tissues, the cervix was severed without the loss of a single drop of blood. The operation occupied about ten minutes' time. Warm vaginal injections of a weak solution of carbolic acid were ordered three times a day. Her recovery was rapid. The dysmenorrhea and dyspareunia were completely relieved.

A great many writers, chiefest among whom is Dr. T. A. Emmet, claim that amputation of the cervix, especially with the galvano-cautery, is followed by contraction of the resultant cicatricial tissue and closure of the os in most instances. Their statements I do not doubt, but in this case the contraction was toward the centre of the wound surrounding the cervix, which pulled the os open. This I positively know, for not having seen the lady for several months until November 27th, 1879, more than a year after the operation, she was in my office, and I mentioned Dr. Emmet's statement concerning the results of such operations and desired an examination, to which she willingly consented. The cervix extends between one-half and three-quarters of an inch within the vagina, is not enlarged, the os is easily detected with the finger, is wider laterally than antero-posteriorly, readily admits Simpson's sound, which passes two inches and a half in the proper direction for the uterine canal; in fact, except that a faint line of cicatricial tissue can be detected by sight, but not by the touch, surrounding the os, no one could suspect the uterus to have ever been in any but the most healthy condition. There is no flexion or version nor menstrual difficulty.

The battery I use is one of Byrne's perfected, made by the Western Electric Manufacturing Company, 220 Kinzie street, Chicago. It is the smallest efficient battery that is made, and can be easily transported with all necessary accessories in a small wooden valise.

It is an excellent battery for electrolysis also, as I have by that method dissipated growths, but will leave the discussion of that subject for some future paper.

The pipes for blowing the gas off the plates during action are entirely too brittle, but can easily be mended with bits of soft rubber tubing. The handles could be improved by having slide break-circuits applied. The ecraseur is a miserable thing that I do not believe any one would try to work with twice. Every one using the battery ought to have sufficient ingenuity to improvise electrodes. Perhaps I will refer to them at a future time.

No. 407 Jersey Street.

